# P05.22. Health beliefs and experiences of patients with chronic Lyme disease: a qualitative study

**DOI:** 10.1186/1472-6882-12-S1-P382

**Published:** 2012-06-12

**Authors:** A Ali, J Millet, L Vitulano, R Lee, E Colson

**Affiliations:** 1 Yale University School of Medicine, New Haven, USA; 2 National College of Natural Medicine, Portland, USA

## Purpose

Chronic Lyme disease is a term that describes a constellation of persistent symptoms in patients who may or may not have serologic evidence of *Borrelia burgdorferi* infection. Little has been published in the medical literature about patients with chronic Lyme disease or their relationships with healthcare providers. The objective of this study was to gain insights into the health beliefs and experiences of patients with chronic Lyme disease.

## Methods

This was a qualitative, descriptive study in which face-to-face in-depth interviews were conducted with patients who were diagnosed with or self-identify as having chronic Lyme disease. Patients were recruited through Connecticut-based Lyme disease mailing lists and support groups. A coding structure was developed using an iterative process. Transcribed interviews were coded using Atlas.ti software and analyzed for emergent topics and themes. Interviews were conducted until thematic saturation was achieved.

## Results

A total of 12 interviews were conducted. Four major themes emerged. Patients reported: (1) diminished health status associated with chronic Lyme disease; (2) concerns about persistence of symptoms (e.g., that full recovery was unlikely); (3) two divergent types of physician-patient relationships (i.e., exceptionally supportive or uncaring and dismissive); and (4) seeking and receiving unconventional care (e.g., complementary/alternative therapies and/or prolonged treatment with antibiotics).

## Conclusion

Our findings show that patients report a marked decrease in health status associated with chronic Lyme disease and are often unsatisfied with care in conventional settings. Negative experiences with providers were associated with reports of dismissive, patronizing, or condescending attitudes. Positive experiences were associated with providers reported to be attentive, optimistic, and supportive. Patient-centered approaches that acknowledge suffering and focus on continuity of care and symptomatic relief may result in increased patient satisfaction.

